# Comparative Evaluation of Endotoxin Activity Level and Various Biomarkers for Infection and Outcome of ICU-Admitted Patients

**DOI:** 10.3390/biomedicines7030047

**Published:** 2019-06-29

**Authors:** Toshiaki Ikeda, Hidenobu Kamohara, Shingo Suda, Takeo Nagura, Mikiko Tomino, Masatoshi Sugi, Zen’ichiro Wajima

**Affiliations:** 1 Division of Critical Care and Emergency Medicine, Tokyo Medical University Hachioji Medical Center, Tokyo 193-0998, Japan; 2 Department of Anesthesiology, Tokyo Medical University Hachioji Medical Center, Tokyo 193-0998, Japan

**Keywords:** blood endotoxin, endotoxin activity assay (EAA), procalcitonin (PCT), presepsin, interleukin (IL)-6, sepsis

## Abstract

Here, we concurrently measured the endotoxin activity (EA) level and levels of multiple biomarkers in patient blood obtained within 24 h after being admitted into the intensive care unit (ICU) and analyzed whether there were links between these markers and their associations with patient conditions and outcomes. The EA levels highly correlated with disease severity and patient survival, and showed a significant positive association with levels of lactate, procalcitonin, presepsin, and interleukin-6. Notably, the EA level was the marker that most highly correlated with the results of blood culture, and the presepsin level was the marker most highly correlated with the survival outcome at 28 days. Thus, the optimal biomarker should be selected based on whether it will be used to discriminate the presence of an infection or to predict survival.

## 1. Introduction

Endotoxin, a membrane component of Gram-negative bacteria, plays a key role in the pathogenesis of sepsis [1,2]. Endotoxin flows into the blood due to bacterial infection or bacterial translocation, associated with a failure of the intestinal barrier and causes various inflammatory responses.

The limulus amebocyte lysate (LAL) test is the most widely used test to detect and quantify bacterial endotoxins, however endotoxin activity assay (EAA), which is a rapid measurement method using whole blood, has become widespread in recent years [3].

In addition to endotoxin, various blood biomarkers have previously been evaluated for the purpose of diagnosing sepsis or predicting its prognosis. For example, lactate levels can be used as a marker for systemic tissue hypoperfusion in patients with circulatory shock. It is now included in the clinical criteria for septic shock defined in the Third International Consensus Definition for Sepsis and Septic Shock (Sepsis-3) [4]. White blood cell count (WBC) and C-reactive protein (CRP) are widely used as clinical markers of inflammation. In addition, procalcitonin (PCT) has been put into practical use as diagnostic agents for sepsis [5]. Although not as widely used in routine clinical settings, presepsin and cytokines such as interleukin (IL)-6 have also been demonstrated as useful for the diagnosis and evaluation of sepsis [6,7,8,9]. The characteristics and usefulness of these biomarkers are compared in many studies [10]. However, a detailed comparison between EA levels and these biomarkers are scarcely reported.

Here, we concurrently measured the levels of endotoxin activity (EA) and multiple biomarkers in a patient’s blood that was obtained within 24 h after ICU admission and analyzed: (1) Whether there were associations between these markers, and (2) the usefulness of each biomarker in detecting infection and predicting patient outcomes.

## 2. Experimental Section

### 2.1. Patient Characteristics

A total of 142 patients diagnosed with sepsis or suspected sepsis and admitted to the ICU at our hospital between January 2014 and June 2018 were included in this study. After excluding the 13 patients missing EAA data, 129 cases were analyzed. The EA level and the levels of multiple biomarkers were measured concurrently using samples of patient blood obtained within 24 h after ICU admission. This study was conducted with the approval of the institutional review board of Tokyo Medical University Hachioji Medical Center (No.H-122, approval date: 20 January 2009. For inclusion in this study, written informed consent was obtained from each patient. If the patients did not have the capacity to provide consent, their consent was obtained from their legal representatives.

### 2.2. Endotoxin Activity Assay (EAA)

The EA level in whole blood was measured using an EAA in accordance with the assay protocol recommended by the manufacturer (Spectral Diagnostics, Canada). The EAA is a diagnostic test that uses an anti-endotoxin monoclonal antibody that measures the reaction of neutrophils to endotoxin–anti-endotoxin complexes, the amount of which depends on the amount of endotoxin in the blood sample [11]. The EAA received Food and Drug Administration (FDA) clearance in 2003 following the MEDIC clinical trial, which measured EA levels of critically ill patients admitted to the ICU and demonstrated that EA levels correlated with a higher risk of developing sepsis, as well as with higher mortality [12]. EAA specifications did not change during the 4-year study period. Whole blood was taken from the patients, collected into a conventional blood collection tube (Terumo Venoject II, Terumo, Japan) containing ethylenediaminetetraacetic acid (EDTA) as an anticoagulant and incubated for 10 min at 37 °C. For each sample, 40 µL was incubated in duplicate with saturating concentrations (2.6 µg/mL) of endotoxin-specific anti-lipid A antibody (IgM monoclonal antibody) in 1 mL of Hank’s balanced salt solution buffer containing heparin. This combination allowed the formation of endotoxin–anti-endotoxin antibody complexes and complement proteins opsonized these complexes. The opsonized immune complexes prime neutrophils in the blood enhanced the respiratory burst in response to zymosan in the assay reagent, yielding oxidants that reacted with luminol in the reaction mixture to emit chemiluminescence. Chemiluminescence was detected in a photon-counting luminometer (Berthold Detection Systems, Pforzheim, Germany). A basal activity in the absence of antibody (negative control tube) and the maximum respiratory burst activity in the presence of excess (4600 pg/mL) exogenous endotoxin spiked into the blood sample (positive control tube) were measured in parallel. The EA level was calculated by the following equation:

EA level = (sample tube − negative control tube)/(positive control tube − negative control tube)(1)

The assay was conducted within 30 min of blood sample collection. All measurements were done in duplicate.

### 2.3. Laboratory Analyses

EDTA-plasma PCT levels were measured using an electrochemiluminescence immunoassay (ECLIA) kit (Elecsys BRAHMS PCT, BRAHMS, Germany) in accordance with the assay protocol recommended by the manufacturer. Values under the detection limit (0.1 ng/mL) were recorded as 0.1 for the association analysis. The presepsin concentration was measured in EDTA-plasma with a compact automated immunoanalyzer, PATHFAST (LSI Medience Co., Japan), in accordance with the assay protocol recommended by the manufacturer, which was based on a chemiluminescent enzyme immunoassay (CLEIA). Lactate concentrations in whole blood were measured with Radiometer ABL800 FLEX (Radiometer, Denmark), and IL-6 concentrations were measured using serum samples by a CLEIA system (Human IL-6 CLEIA Fujirebio, Fujirebio, Japan) in accordance with each assay protocol recommended by the manufacturer. To collect samples, we used conventional blood collection tubes without anticoagulant for IL-6 measurement and tubes containing EDTA for PCT and presepsin measurement.

### 2.4. Statistical Analysis

The data were analyzed by a Jonckheere–Terpstra test and Mann-Whitney U test using EZR (Easy R, Saitama Medical Center, Jichi Medical University, Japan), with values of *p* < 0.05 considered statistically significant.

## 3. Results

### 3.1. Patient Characteristics

In total, 129 patients admitted to the ICU were enrolled in the study. The clinical characteristics and etiology of infection of these patients are shown in Table 1. The median age of the patients was 71 years (interquartile range (IQR): 66–77), and 92 patients (71.3%) were male. The median Acute Physiology and Chronic Health Evaluation II (APACHE II) score was 23 (IQR: 16–30), and the median Sequential Organ Failure Assessment (SOFA) score was 9 (IQR: 5–12). The mortality rate was 22.5% (29/129) at 28 days after ICU admission.

### 3.2. Comparison Between the EA Level and Disease Severity or Mortality

**Figure 1:**
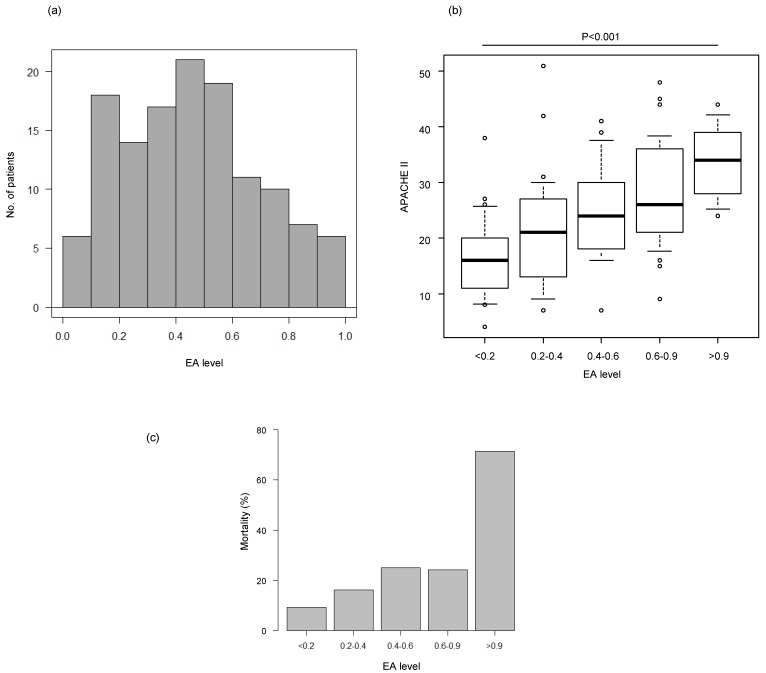
(**a**) Distribution of patient endotoxin activity (EA) levels. (**b**) Patient APACHE II scores stratified by patient EA levels. The data were analyzed by a Jonckheere–Terpstra test after an analysis by a Kruskal–Wallis test (*p* < 0.0001). Significant differences (*p* < 0.001) in APACHE II were observed between EA levels. Box plots indicate 75th percentile (top edge of box), 25th percentile (bottom edge of box), and 50th percentile (bold horizontal line in box); whiskers indicate 90th percentile (top) and 10th percentile (bottom); circles indicate values that exceed 10th to 90th percentile range. (**c**) Mortality stratified by patient EA levels. The data were analyzed by a Fisher’s exact test and significant differences (*p* = 0.0225) in mortality were observed between EA levels.

Figure 1 shows the distribution of the EA levels of all ICU-admitted patients and patient APACHE II scores or 28-day mortality stratified by patient EA levels. Patients were divided into five groups based on their EA levels: 1) Less than 0.2 (<0.2); 2) equal to or higher than 0.2 and lower than 0.4 (0.2–0.4); 3) equal to or higher than 0.4 and lower than 0.6 (0.4–0.6); 4) equal to or higher than 0.6 and lower than 0.9 (0.6–0.9); and 5) equal to or higher than 0.9 (>0.9). Although the EA levels of most patients were in the 0.4–0.6 range, these levels were widely distributed in the range of 0–1 (Figure 1A). The APACHE II score increased as the EA level increased. The median APACHE II score was 16 (IQR: 11–20) in the <0.2 group, whereas it was 34 (IQR: 28–39) in the >0.9 group (Figure 1B). Twenty-eight-day mortality also increased as the EA level increased, with a mortality rate of 9.1% (2/22) in the <0.2 group and a mortality rate of 71.4% (5/7) in the >0.9 group (Figure 1C).

### 3.3. Comparison Between the EA Level and Various Biomarkers

**Figure 2:**
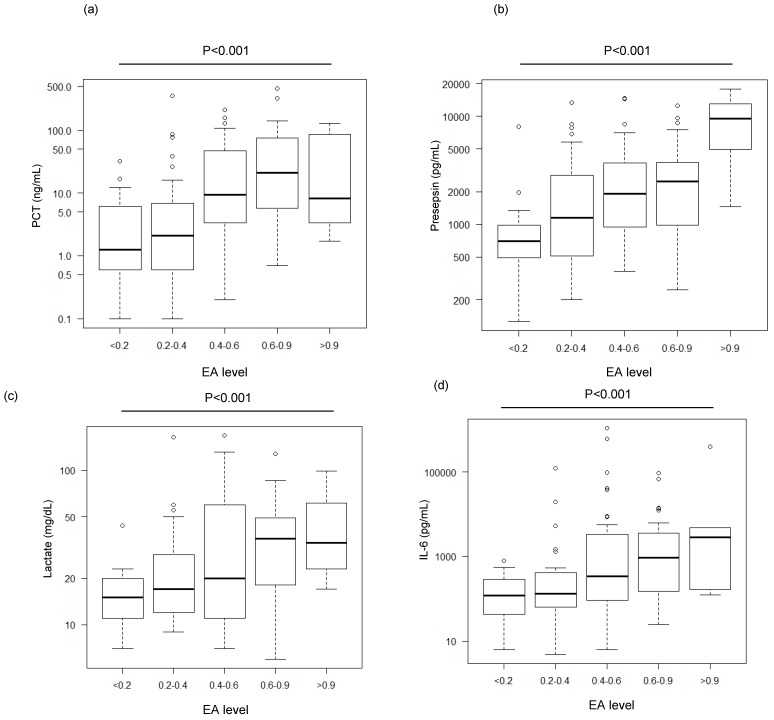
Distribution of various biomarkers’ levels stratified by EA levels. (**a**) procalcitonin (PCT), (**b**) presepsin, (**c**) lactate, and (**d**) interleukin (IL)-6. The data were analyzed by a Jonckheere–Terpstra test after analysis by a Kruskal–Wallis test, which showed significant differences ((**a**), (**b**) *p* < 0.0001, (**c**) *p* = 0.0154, (**d**) *p* = 0.0046). Significant differences (*p* < 0.001) in all biomarkers were observed between EA levels.

Figure 2 shows the distribution of the levels of various biomarkers stratified by EA levels. The level of each measured biomarker trended higher as the EA value increased. For example, the median presepsin level was 9487 (IQR: 5240–12,907) pg/mL in the >0.9 group, a level which is more than three times higher than that of the 0.6–0.9 group (2487 (983–3770) pg/mL).

### 3.4. Comparison Between Blood Culture Results and Various Biomarkers

Next, to further investigate the characteristics of each biomarker, we analyzed whether there were associations between the blood culture results and the EA, PCT, presepsin, and IL-6 levels.

**Figure 3:**
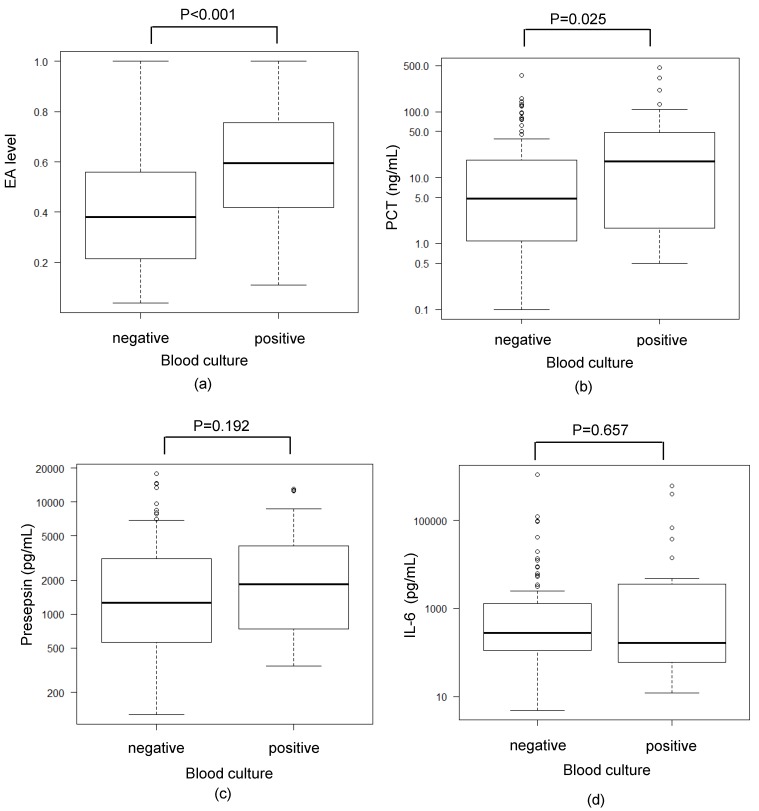
Comparison between the blood culture results and various biomarkers. (**a**–**d**) Comparisons between the EA level (**a**), PCT level (**b**), presepsin level (**c**), or IL-6 level (**d**) in the blood culture-negative and blood culture-positive groups. The data were analyzed by a Mann–Whitney U test. Significant differences ((**a**) *p* < 0.001, (**b**) *p* = 0.025) in EA level and PCT were observed between groups.

For EA and PCT, the levels in the blood culture-positive group were significantly higher than those in the blood culture-negative group. The median EA level was 0.60 (IQR 0.42–0.75) in the blood culture-positive group, which is significantly higher than the EA level of 0.38 (IQR 0.22–0.56) in the culture-negative patients (*p* < 0.001). In contrast, there were no significant differences among the levels of presepsin and IL-6, as well as between the blood culture-positive and the blood culture-negative groups (Figure 3).

### 3.5. Comparison Between Mortality and Various Biomarkers

**Figure 4:**
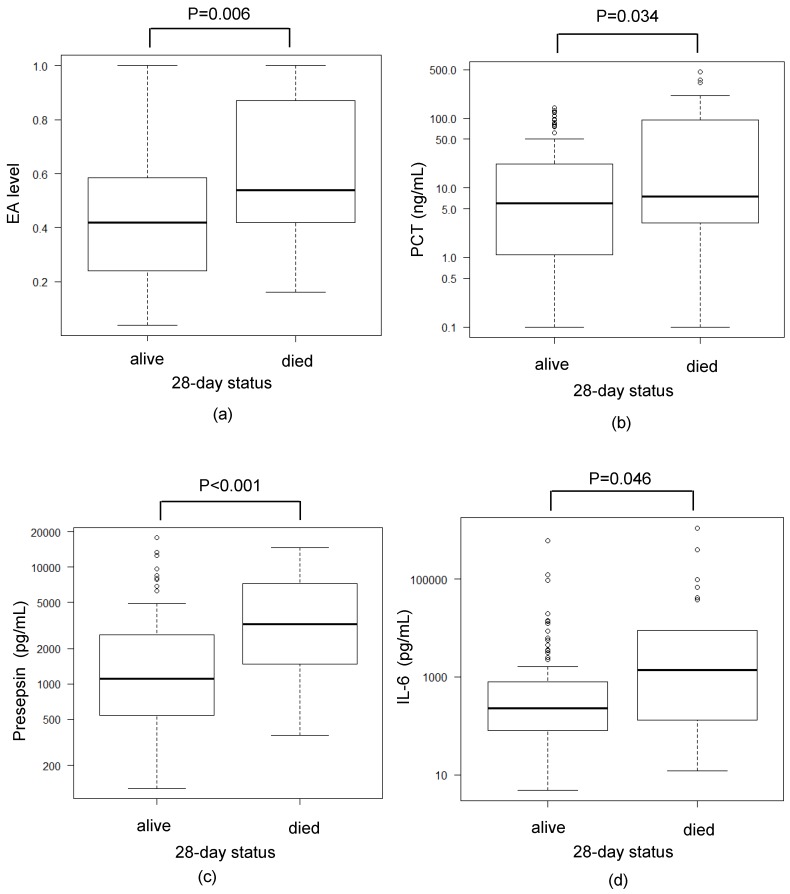
Comparison between mortality and various biomarkers. (**a**–**d**) Comparisons between the EA level (**a**), PCT level (**b**), presepsin level (**c**), and IL-6 level (**d**) in the surviving patients and non-surviving patients at 28 days. The data were analyzed by a Mann–Whitney U test. Significant differences ((**a**) *p* = 0.006 (**b**) *p* < 0.034, (**c**) *p* < 0.001, (**d**) *p* = 0.046) in all biomarkers were observed between groups.

Regarding associations with the patient outcome after 28 days, the levels of all measured biomarkers were significantly higher in non-surviving patients. Most strikingly, the median level of presepsin was 1108 (IQR: 550–2623) pg/mL in the survivor group, which was remarkably lower than the presepsin level of 3251 (IQR: 1486–7254) pg/mL in the non-survivor group (Figure 4).

### 3.6. Association Between Infecting Organisms and EAA and PCT Levels

**Figure 5:**
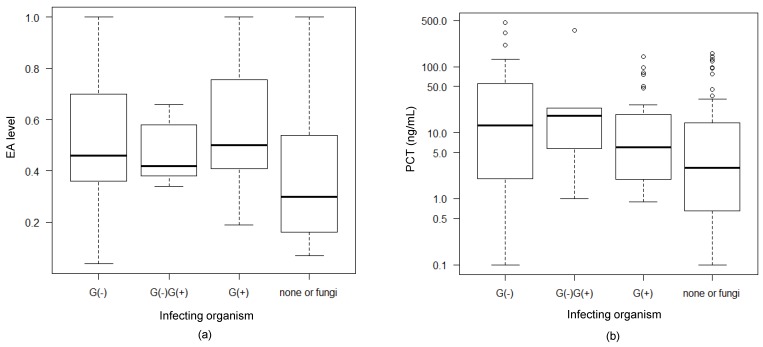
Association between the infecting organisms and biomarkers. (**a**,**b**) Comparisons between the EA level (**a**) and PCT level (**b**) among various types of infecting organisms: Gram-negative bacteria alone [G(-)], a mixture of both Gram-negative and Gram-positive bacteria [G(-)G(+)], Gram-positive bacteria alone [G(+)], and other (none or fungi). The data were analyzed by a Mann–Whitney U test. PCT levels tended to be higher in patients infected with Gram-negative bacteria, whereas EA levels did not show any detectable difference between patients infected with Gram-negative or Gram-positive bacteria.

For EA and PCT levels, we examined their associations with the infecting organism (Figure 5). PCT levels tended to be higher in patients infected with Gram-negative bacteria, whereas EA levels did not show any detectable difference between patients infected with Gram-negative or Gram-positive bacteria.

## 4. Discussion

In this study, we measured EA levels using an EAA, as well as the levels of several biomarkers, in the blood of patients with sepsis or suspected sepsis that were admitted to an ICU in Japan. The results indicate that EA levels highly correlated with disease severity and patient outcome and that EA levels showed a significant positive association with levels of lactate, PCT, presepsin, and IL-6. Additionally, the EA level was the marker that most highly correlated with results of blood culture, and presepsin was the marker that most highly correlated with the patient outcome at 28 days.

Each tested biomarker exhibited different characteristics, which likely reflect the differences in mechanism by which the levels of these markers arose. During inflammation, PCT is produced mainly by two alternative mechanisms: 1) The direct pathway induced by lipopolysaccharide (LPS) and other toxic metabolites from microbes and 2) the indirect pathway induced by various inflammatory mediators like IL-6 and TNF-α [13]. A circulating PCT level is reported to be a clinically useful marker for the diagnosis of sepsis and the prediction of ICU patient outcomes, as well as a guide to the initiation and termination of antibiotics [14,15,16,17]. Presepsin is produced mainly by a mechanism that is related to bacterial phagocytosis, and the cleavage of membrane CD14 by lysosomal enzymes of granulocytes is involved in its secretion [18]. In contrast, IL-6 is regarded as a nonspecific inflammatory marker and has low specificity for infection [19], and our results are in accordance with these previous findings. The timing of each biomarker increase is also different. IL-6 mediate the initial innate immune system response to injury or infection and its plasma concentration increases and falls rapidly. On the other hand, PCT level goes up after the IL-6 level reaches peak and the high concentration continues longer than IL-6 [20].

Characteristics of each biomarker have been studied in various different types of patients. For example, in the patients with organ dysfunction in the ICU, serum IL-6 levels had the highest diagnostic value for infection among IL-6, PCT, presepsin, and CRP levels [21]. In patients with severe trauma, elevated plasma concentrations of presepsin in the clinical course were associated with the presence of systemic inflammatory response syndrome (SIRS), whereas PCT and IL-6 did not [22]. Overall, optimal biomarkers should be selected based on their intended purpose, such as to discriminate between infected and uninfected patients, or to predict patient survival.

We found that the EA level highly correlated with the presence or absence of infection, but there was no difference in the EA level between Gram-negative bacterial infection and Gram-positive bacterial infection. Although it is not clear why the EA level was also high in patients infected with Gram-positive bacteria, which do not produce endotoxin, it is possible that endotoxin in the blood may rise regardless of the infecting bacterial species due to bacterial translocation associated with the failure of the intestinal barrier that occurs under severe conditions. In fact, EA levels are reported to increase in multiple trauma patients and post-cardiac arrest shock patients in parallel with the damage of intestinal barrier [23,24]. Alternately, the EA level may be influenced by the state of neutrophils, because the EA level is calculated from a measurement of reactive oxygen produced by neutrophils in a specimen stimulated by an endotoxin complex produced using an anti-endotoxin antibody as a reagent. In contrast, the level of PCT was higher in patients infected with Gram-negative bacteria compared with those infected with Gram-positive bacteria, and this result is consistent with previous reports [25].

Polymyxin B hemoperfusion is a blood purification device which removes circulating endotoxins from patient blood. It is used as the adjunctive therapy of septic shock [26]. In the latest clinical study evaluating this therapy, the EUPHRATES trial conducted in North America, EAA was used as part of the entry criteria for patients [27]. In the EUPHRATES trial evaluating the effectiveness of polymyxin B hemoperfusion, which removes endotoxin by extracorporeal circulation, EAA was used as part of the entry criteria for patients [27]. In the EUPHRATES trial, patients with an EA level of 0.6 or higher were included, however, our results show that patients with intermediate EA levels (0.4–0.6) were also critically ill and had a mortality equivalent to that of patients in the high EA level group (0.6–0.9). In our previous study, which measured the EA levels of Japanese healthy volunteers, the EA levels were distributed in the lower range compared with the results of the MEDIC study, which measured the EA levels of Westerners [28]. Whether the distribution of EA levels in sepsis patients also differs by race or region is unknown, but our results suggest that there is a possibility that Japanese patients exhibit lower EA levels than Westerners when the disease severities are similar. We speculate that, at least in Japan, patients with EA levels between 0.4 and 0.6 may also be suitable subjects for endotoxin removal therapy.

In the post-hoc analysis of the EUPHRATES trial, it was reported that the survival benefit was obtained by targeting patients with 0.6 ≤ EA < 0.9, excluding patients with an EA level of ≥0.9 [29]. In our study, although the frequency of patients with an EA level of 0.9 or higher (>0.9 group) was only about 5% of the total, the APACHE II score in this group was remarkably high, with a median value of 34. The mortality rate of this group was also high (71.4%), compared with the 0.6–0.9 group (24.1%). For patients with EA levels higher than 0.9, the removal of endotoxin by blood purification may have failed because the disease severity was too high. In this study, we analyzed whether there were associations between the EA level and various biomarkers, and found that presepsin highly correlated with the EA level. The level of presepsin was very high (9487 pg/mL) in the >0.9 group. For the purpose of excluding patients corresponding to EA ≥ 0.9 in the EUPHRATES trial, which were patients who did not benefit from endotoxin removal therapy, it might be possible to use presepsin as an alternative marker. For example, presepsin levels higher than 10,000 pg/mL could be used as a cutoff to distinguish patients who are too sick and are not a suitable target for endotoxin removal therapy. A prospective study is needed to confirm this possibility in the future.

Our study has several limitations. It was a single-site, retrospective, observational study, and the number of patients was relatively small. The EAA measurement and collection of specimens for each marker measurement were performed within 24 h of each patient entering the ICU, but the exact timing was not unified. Additionally, biomarker measurements for some patients were not available.

## 5. Conclusions

The level of EA showed a high association with patient disease severity and survival, and it also showed a significant association with the levels of lactate, PCT, presepsin, and IL-6. The EA level was the tested marker that most strongly correlated with the results of blood culture, and the presepsin level was the tested marker that most strongly correlated with patient outcome at 28 days. Thus, it is important to select the optimal marker for evaluation according to the intended purpose, such as to discriminate whether an infection is present or to predict patient outcome.

## Figures and Tables

**Table 1:** Characteristics of the study population.

Parameter	Value
Age (median (IQR))	71 (66–77)
Male (*n* (%))	92 (71.3%)
Female (*n* (%))	37 (28.7%)
Severity (median (IQR))	
APACHE II score	23 (16–30)
SOFA score	9 (5–12)
Blood culture (*n* (%))	
Positive	37 (28.7%)
Negative	92 (71.3%)
Infecting organism (*n* (%))	
Gram-negative	41 (31.8%)
Gram-positive	26 (20.2%)
Mixed infection	5 (3.9%)
Other (None or fungi)	57 (44.2%)
28-day mortality (*n* (%))	29 (22.5%)

For quantitative variables, median and range (in parenthesis) are given. Values are presented as No. (%) or median (interquartile (IQR) range). APACHE II, Acute Physiology and Chronic Health Evaluation II; SOFA, Sequential Organ Failure Assessment.

## Abbreviations

EA level	endotoxin level
ICU	intensive care unit
EAA	endotoxin activity assay
PCT	procalcitonin
IL-6	interleukin-6
LAL	limulus amebocyte lysate
WBC	white blood cell count
CRP	C-reactive protein
FDA	Food and Drug Administration
EDTA	ethylenediaminetetraacetic acid
IQR	interquartile range
ECLIA	electrochemiluminescence immunoassay
CLEIA	chemiluminescent enzyme immunoassay
APACHE II	Acute Physiology and Chronic Health Evaluation II
SOFA	Sequential Organ Failure Assessment
SIRS	systemic inflammatory response syndrome
